# Supplementary material for: Naturally acquired antibodies against 4 Streptococcus pneumoniae serotypes in Pakistani adults with type 2 diabetes mellitus
Source: PLoS One. 2024 Aug 9;19(8):e0306921. doi: 10.1371/journal.pone.0306921 (PMC11315336; doi:10.1371/journal.pone.0306921)
Supplement: S1 Table — (DOCX) [file pone.0306921.s001.docx]

| Lab ID  **S1 Table.** Serotype-specific IgG concentration values of participants | Serotype19F IgG (µg/mL) | Serotype 1 IgG (µg/mL) | Serotype 18C IgG (µg/mL) | Serotype 9V IgG (µg/mL) |
| --- | --- | --- | --- | --- |
| 1 | 2.033 | 1.453 | 2.705 | 1.916 |
| 2 | 18.844 | 0.945 | 4.944 | 4.921 |
| 3 | 6.602 | 1.637 | 2.53 | 1.511 |
| 4 | 20.747 | 1.779 | 8.601 | 3.393 |
| 5 | 3.387 | 0.6 | 5.002 | 1.391 |
| 6 | 9.304 | 1.968 | 3.989 | 2.295 |
| 7 | 13.92 | 3.895 | 2.53 | 1.92 |
| 8 | 4.393 | 1.731 | 4.232 | 2.382 |
| 9 | 3.401 | 0.635 | 1.97 | 1.226 |
| 10 | 36.765 | 2.849 | 10.32 | 15.949 |
| 11 | 6.125 | 0.861 | 0.972 | 0.964 |
| 12 | 5.521 | 1.121 | 17.228 | 2.991 |
| 13 | 2.262 | 0.859 | 4.459 | 18.602 |
| 14 | 7.437 | 3.249 | 8.462 | 14.259 |
| 15 | 15.088 | 1.456 | 4.522 | 7.436 |
| 16 | 3.322 | 0.466 | 3.005 | 1.39 |
| 17 | 21.2 | 3.762 | 5.455 | 5.28 |
| 18 | 11.192 | 1.14 | 3.864 | 4.061 |
| 19 | 3.53 | 0.708 | 3.514 | 0.748 |
| 20 | 3.84 | 1.241 | 2.412 | 1.44 |
| 21 | 3.007 | 1.18 | 2.218 | 1.035 |
| 22 | 10.998 | 0.865 | 4.915 | 2.927 |
| 23 | 5.522 | 1.612 | 3.256 | 1.864 |
| 24 | 3.6 | 0.547 | 3.889 | 1.875 |
| 25 | 10.17 | 2.816 | 3.657 | 5.662 |
| 26 | 6.621 | 0.769 | 5.112 | 2.195 |
| 27 | 1.976 | 1.091 | 3.625 | 2.329 |
| 28 | 5.552 | 1.836 | 3.103 | 3.143 |
| 29 | 1.884 | 0.619 | 2.812 | 1.038 |
| 30 | 6.182 | 1.185 | 1.909 | 1.723 |
| 31 | 2.308 | 0.531 | 3.387 | 0.557 |
| 32 | 2.749 | 0.73 | 1.593 | 0.768 |
| 33 | 5.739 | 1.087 | 4.721 | 2.809 |
| 34 | 9.964 | 1.553 | 5.604 | 12.311 |
| 35 | 9.295 | 5.889 | 4.53 | 4.513 |
| 36 | 36.932 | 3.421 | 13.209 | 8.632 |
| 37 | 2.828 | 0.024 | 1.905 | 0.772 |
| 38 | 22.097 | 3.437 | 7.04 | 3.73 |
| 39 | 9.905 | 1.73 | 4.859 | 4.775 |
| 40 | 10.741 | 2.054 | 12.446 | 3.943 |
| 41 | 2.9 | 2.153 | 1.459 | 1.101 |
| 42 | 5.491 | 3.611 | 4.781 | 7.964 |
| 43 | 13.709 | 1.354 | 2.415 | 1.789 |
| 44 | 22.331 | 3.94 | 7.457 | 19.504 |
| 45 | 6.587 | 1.441 | 2.72 | 3.585 |
| 46 | 21.12 | 0.801 | 2.734 | 1.44 |
| 47 | 2.134 | 0.865 | 2.7 | 0.797 |
| 48 | 4.191 | 1.704 | 9.245 | 13.887 |
| 49 | 15.261 | 2.632 | 7.025 | 9.464 |
| 50 | 3.452 | 0.917 | 6.757 | 1.82 |
| 51 | 3.79 | 0.705 | 1.709 | 1.036 |
| 52 | 10.795 | 1.035 | 5.029 | 13.744 |
| 53 | 7.779 | 2.171 | 3.786 | 9.426 |
| 54 | 7.073 | 1.774 | 4.42 | 2.504 |
| 55 | 2.424 | 0.468 | 1.312 | 2.592 |
| 56 | 11.507 | 2.496 | 12.368 | 10.389 |
| 57 | 1.563 | 0.89 | 2.328 | 1.486 |
| 58 | 19.864 | 1.637 | 9.092 | 6.776 |
| 59 | 28.32 | 2.404 | 11.231 | 9.12 |
| 60 | 3.986 | 3.086 | 14.741 | 4.456 |
| 61 | 19.286 | 2.333 | 12.2 | 4.33 |
| 62 | 4.126 | 1.775 | 6.293 | 1.041 |
| 63 | 26.593 | 3.318 | 13.191 | 16.645 |
| 64 | 17.714 | 0.507 | 1.457 | 1.938 |
| 65 | 5.858 | 0.658 | 4.679 | 1.259 |
| 66 | 7.81 | 1.485 | 5.586 | 2.936 |
| 67 | 6.754 | 1.033 | 5.879 | 4.947 |
| 68 | 24.48 | 0.614 | 5.292 | 3.36 |
| 69 | 20.23 | 1.649 | 5.112 | 6.105 |
| 70 | 19.2 | 2.759 | 10.857 | 9.12 |
| 71 | 14.895 | 2.066 | 4.812 | 2.568 |
| 72 | 1.742 | 0.594 | 0.67 | 0.59 |
| 73 | 1.367 | 0.992 | 11.812 | 1.23 |
| 74 | 4.8 | 1.579 | 3.069 | 13.92 |
| 75 | 6.134 | 1.624 | 16.116 | 6.426 |
| 76 | 8.245 | 1.25 | 4.964 | 3.849 |
| 77 | 12.668 | 3.49 | 10.185 | 10.651 |
| 78 | 4.659 | 1.348 | 6.996 | 1.556 |
| 79 | 5.345 | 0.94 | 5.577 | 11.44 |
| 80 | 6.72 | 2.714 | 3.416 | 9.12 |
| 81 | 10.258 | 0.962 | 1.459 | 1.878 |
| 82 | 8.365 | 1.374 | 6.537 | 5.54 |
| 83 | 3.222 | 0.663 | 4.075 | 1.254 |
| 84 | 3.206 | 1.252 | 2.776 | 0.864 |
| 85 | 9.296 | 1.113 | 2.832 | 1.205 |
| 86 | 3.837 | 0.596 | 2.864 | 1.646 |
| 87 | 11.583 | 1.006 | 20.727 | 2.527 |
| 88 | 36.96 | 6.802 | 6.637 | 26.4 |
| 89 | 2.897 | 2.145 | 1.45 | 1.105 |
| 90 | 13.997 | 1.568 | 4.086 | 1.671 |
| 91 | 33.12 | 2.71 | 9.001 | 6.24 |
| 92 | 5.263 | 1.233 | 10.982 | 1.99 |
| 93 | 17.28 | 4.392 | 4.745 | 9.12 |
| 94 | 12.974 | 1.988 | 3.213 | 1.461 |
| 95 | 4.473 | 0.832 | 3.213 | 1.908 |
| 96 | 2.85 | 0.648 | 2.664 | 0.887 |
| 97 | 8.16 | 5.888 | 5.858 | 6.24 |
| 98 | 9.12 | 0.534 | 1.664 | 1.44 |
| 99 | 2.88 | 1.607 | 3.53 | 10.08 |
| 100 | 36.96 | 2.152 | 3.995 | 4.8 |
| 101 | 2.88 | 0.492 | 2.531 | 2.88 |
| 102 | 3.84 | 3.46 | 3.156 | 1.92 |
| 103 | 4.8 | 1.241 | 1.768 | 1.92 |
| 104 | 10.673 | 1.343 | 9.873 | 2.262 |
| 105 | 2.4 | 0.637 | 2.5 | 1.44 |
| 106 | 21.6 | 1.02 | 2.646 | 3.36 |
| 107 | 12 | 1.235 | 4.534 | 3.36 |
| 108 | 2.88 | 0.666 | 2.002 | 0.48 |
| 109 | 1.92 | 0.685 | 1.352 | 0.96 |
| 110 | 1.92 | 0.734 | 1.476 | 0.96 |
| 111 | 7.68 | 1.216 | 2.424 | 1.44 |
| 112 | 5.76 | 0.858 | 4.509 | 5.28 |
| 113 | 4.32 | 0.736 | 3.487 | 3.84 |
| 114 | 1.44 | 0.556 | 0.693 | 0.48 |
| 115 | 1.44 | 0.413 | 1.389 | 0.48 |
| 116 | 2.746 | 0.732 | 1.598 | 0.769 |
| 117 | 1.44 | 0.413 | 1.389 | 0.48 |
| 118 | 6.72 | 2.095 | 2.918 | 2.4 |
| 119 | 1.205 | 0.501 | 7.486 | 1.008 |
| 120 | 0.96 | 0.738 | 1.812 | 0.96 |
| 121 | 4.039 | 1.334 | 2.196 | 1.841 |
| 122 | 2.88 | 0.66 | 3.052 | 1.92 |
| 123 | 25.92 | 1.181 | 6.988 | 4.32 |
| 124 | 3.84 | 3.24 | 3.319 | 1.92 |
| 125 | 6.3 | 0.246 | 2.613 | 2.973 |
| 126 | 6.499 | 0.271 | 3.799 | 1.579 |
| 127 | 0.96 | 0.474 | 1.172 | 0.48 |
| 128 | 0.618 | 0.261 | 0.872 | 0.379 |
| 129 | 2.486 | 0.24 | 7.689 | 0.737 |
| 130 | 5.28 | 1.158 | 4.752 | 2.88 |
| 131 | 3.84 | 1.75 | 3.701 | 2.88 |
| 132 | 3.36 | 0.949 | 6.843 | 0.96 |
| 133 | 2.88 | 0.289 | 7.392 | 1.257 |
| 134 | 6.157 | 1.146 | 10.727 | 2.226 |
| 135 | 2.181 | 0.224 | 1.717 | 1.671 |
| 136 | 2.127 | 3.687 | 4.803 | 1.677 |
| 137 | 2.88 | 0.639 | 3.178 | 3.36 |
| 138 | 2.629 | 0.448 | 2.089 | 2.724 |
| 139 | 1.76 | 0.62 | 3.266 | 6.541 |
| 140 | 7.2 | 2.328 | 5.732 | 4.32 |
| 141 | 3.297 | 0.781 | 7.48 | 1.775 |
| 142 | 1.054 | 0.315 | 2.018 | 0.887 |
| 143 | 6.404 | 3.371 | 7.841 | 4.547 |
| 144 | 2.275 | 0.537 | 5.807 | 2.746 |
| 145 | 4.436 | 0.615 | 3.308 | 1.175 |
| 146 | 3.043 | 0.436 | 1.769 | 1.976 |
| 147 | 2.4 | 0.709 | 5.025 | 1.44 |
| 148 | 4.65 | 0.503 | 6.326 | 1.073 |
| 149 | 1.889 | 0.79 | 2.928 | 0.822 |
| 150 | 2.88 | 0.71 | 1.461 | 0.48 |
| 151 | 1.44 | 0.564 | 1.462 | 1.44 |
| 152 | 2.88 | 0.412 | 2.846 | 0.96 |
| 153 | 0.96 | 1.346 | 1.155 | 1.92 |
| 154 | 8.718 | 1.023 | 5.076 | 3.58 |
| 155 | 4.32 | 0.62 | 2.214 | 2.88 |
| 156 | 1.144 | 1.182 | 2.65 | 1.095 |
| 157 | 4.32 | 1.536 | 4.27 | 2.88 |
| 158 | 1.44 | 0.797 | 1.479 | 1.92 |
| 159 | 6.24 | 0.754 | 1.786 | 1.44 |
| 160 | 5.28 | 0.451 | 1.817 | 1.92 |
| 161 | 4.999 | 0.6 | 3.121 | 2.524 |
| 162 | 8.652 | 0.78 | 7.893 | 6.95 |
| 163 | 74.222 | 1.416 | 3.559 | 1.597 |
| 164 | 2.88 | 1.785 | 3.235 | 1.92 |
| 165 | 7.872 | 1.831 | 2.937 | 2.058 |
| 166 | 2.4 | 0.685 | 3.208 | 7.68 |
| 167 | 5.171 | 0.331 | 2.609 | 2.202 |
| 168 | 2.901 | 0.7 | 2.559 | 1.652 |
| 169 | 1.919 | 1.788 | 1.872 | 0.769 |
| 170 | 3.84 | 1.721 | 3.88 | 7.121 |
| 171 | 0.597 | 0.568 | 1.826 | 0.38 |
| 172 | 2.562 | 0.305 | 1.58 | 1.203 |
| 173 | 0.613 | 0.253 | 0.86 | 0.373 |
| 174 | 14.959 | 2.365 | 4.302 | 3.4 |
| 175 | 6.438 | 0.729 | 4.849 | 3.346 |
| 176 | 6.05 | 10.846 | 8.034 | 7.213 |
