# Supplementary material for: Naturally acquired antibodies against 4 Streptococcus pneumoniae serotypes in Pakistani adults with type 2 diabetes mellitus
Source: PLoS One. 2024 Aug 9;19(8):e0306921. doi: 10.1371/journal.pone.0306921 (PMC11315336; doi:10.1371/journal.pone.0306921)
Supplement: S2 Table — (DOCX) [file pone.0306921.s002.docx]

| DM-S1  **S2 Table.** Serotype-specific IgG concentration values of those with and without type 2 diabetes  IgG (µg/mL) | DM-19F  IgG (µg/mL) | DM-9V  IgG (µg/mL) | DM-18C IgG(µg/mL) | NDM-S1  IgG (µg/mL) | NDM-19F  IgG (µg/mL) | NDM-9V  IgG (µg/mL) | NDM-18C IgG (µg/mL) |
| --- | --- | --- | --- | --- | --- | --- | --- |
| 1.453 | 2.033 | 1.916 | 2.705 | 0.635 | 3.401 | 1.226 | 1.97 |
| 0.945 | 18.844 | 4.921 | 4.944 | 0.861 | 6.125 | 0.964 | 0.972 |
| 1.637 | 6.602 | 1.511 | 2.53 | 3.249 | 7.437 | 14.259 | 8.462 |
| 1.779 | 20.747 | 3.393 | 8.601 | 1.456 | 15.088 | 7.436 | 4.522 |
| 0.6 | 3.387 | 1.391 | 5.002 | 1.14 | 11.192 | 4.061 | 3.864 |
| 1.968 | 9.304 | 2.295 | 3.989 | 1.18 | 3.007 | 1.035 | 2.218 |
| 3.895 | 13.92 | 1.92 | 2.53 | 1.612 | 5.522 | 1.864 | 3.256 |
| 1.731 | 4.393 | 2.382 | 4.232 | 1.185 | 6.182 | 1.723 | 1.909 |
| 2.849 | 36.765 | 15.949 | 10.32 | 0.531 | 2.308 | 0.557 | 3.387 |
| 1.121 | 5.521 | 2.991 | 17.228 | 0.73 | 2.749 | 0.768 | 1.593 |
| 0.859 | 2.262 | 18.602 | 4.459 | 3.437 | 22.097 | 3.73 | 7.04 |
| 0.466 | 3.322 | 1.39 | 3.005 | 1.73 | 9.905 | 4.775 | 4.859 |
| 3.762 | 21.2 | 5.28 | 5.455 | 2.153 | 2.9 | 1.101 | 1.459 |
| 0.708 | 3.53 | 0.748 | 3.514 | 3.611 | 5.491 | 7.964 | 4.781 |
| 1.241 | 3.131 | 1.119 | 2.293 | 1.354 | 13.709 | 1.789 | 2.415 |
| 0.865 | 10.998 | 2.927 | 4.915 | 0.917 | 3.452 | 1.82 | 6.757 |
| 0.547 | 3.6 | 1.875 | 3.889 | 0.705 | 3.79 | 1.036 | 1.709 |
| 2.816 | 10.17 | 5.662 | 3.657 | 1.774 | 7.073 | 2.504 | 4.42 |
| 0.769 | 6.621 | 2.195 | 5.112 | 2.404 | 28.32 | 9.12 | 11.231 |
| 1.091 | 1.976 | 2.329 | 3.625 | 0.992 | 1.367 | 1.23 | 11.812 |
| 1.836 | 5.552 | 3.143 | 3.103 | 1.624 | 6.134 | 6.426 | 16.116 |
| 0.619 | 1.884 | 1.038 | 2.812 | 2.714 | 6.72 | 9.12 | 3.416 |
| 1.087 | 5.739 | 2.809 | 4.721 | 1.343 | 10.673 | 2.262 | 9.873 |
| 1.553 | 9.964 | 12.311 | 5.604 | 0.637 | 2.4 | 1.44 | 2.5 |
| 5.889 | 9.295 | 4.513 | 4.53 | 0.666 | 2.88 | 0.48 | 2.002 |
| 3.421 | 36.932 | 8.632 | 13.209 | 0.858 | 5.76 | 5.28 | 4.509 |
| 0.024 | 2.828 | 0.772 | 1.905 | 0.413 | 1.44 | 0.48 | 1.389 |
| 2.054 | 10.741 | 3.943 | 12.446 | 2.095 | 6.72 | 2.4 | 2.918 |
| 3.94 | 22.331 | 19.504 | 7.457 | 0.501 | 1.205 | 1.008 | 7.486 |
| 1.441 | 6.587 | 3.585 | 2.72 | 1.334 | 4.039 | 1.841 | 2.196 |
| 0.801 | 21.12 | 1.44 | 2.734 | 0.246 | 6.3 | 2.973 | 2.613 |
| 0.865 | 2.134 | 0.797 | 2.7 | 0.271 | 6.499 | 1.579 | 3.799 |
| 1.704 | 4.191 | 13.887 | 9.245 | 0.24 | 2.486 | 0.737 | 7.689 |
| 2.632 | 15.261 | 9.464 | 7.025 | 1.158 | 5.28 | 2.88 | 4.752 |
| 1.035 | 10.795 | 13.744 | 5.029 | 1.75 | 3.84 | 2.88 | 3.701 |
| 2.171 | 7.779 | 9.426 | 3.786 | 1.536 | 4.32 | 2.88 | 4.27 |
| 0.468 | 2.424 | 2.592 | 1.312 | 0.797 | 1.44 | 1.92 | 1.479 |
| 2.496 | 11.507 | 10.389 | 12.368 | 0.754 | 6.24 | 1.44 | 1.786 |
| 0.89 | 1.563 | 1.486 | 2.328 | 0.451 | 5.28 | 1.92 | 1.817 |
| 1.637 | 19.864 | 6.776 | 9.092 | 0.6 | 4.999 | 2.524 | 3.121 |
| 3.086 | 3.986 | 4.456 | 14.741 | 0.78 | 8.652 | 6.95 | 7.893 |
| 2.333 | 19.286 | 4.33 | 12.2 | 1.416 | 74.222 | 1.597 | 3.559 |
| 1.775 | 4.126 | 1.041 | 6.293 | 1.785 | 2.88 | 1.92 | 3.235 |
| 3.318 | 26.593 | 16.645 | 13.191 | 1.831 | 7.872 | 2.058 | 2.937 |
| 0.507 | 17.714 | 1.938 | 1.457 | 1.721 | 3.84 | 7.121 | 3.88 |
| 0.658 | 5.858 | 1.259 | 4.679 | 0.568 | 0.597 | 0.38 | 1.826 |
| 1.485 | 7.81 | 2.936 | 5.586 | 0.253 | 0.613 | 0.373 | 0.86 |
| 1.033 | 6.754 | 4.947 | 5.879 | 2.365 | 14.959 | 3.4 | 4.302 |
| 0.614 | 24.48 | 3.36 | 5.292 | 0.729 | 6.438 | 3.346 | 4.849 |
| 1.649 | 20.23 | 6.105 | 5.112 | 10.846 | 6.05 | 7.213 | 8.034 |
| 2.759 | 19.2 | 9.12 | 10.857 |  |  |  |  |
| 2.066 | 14.895 | 2.568 | 4.812 |  |  |  |  |
| 0.594 | 1.742 | 0.59 | 0.67 |  |  |  |  |
| 1.579 | 4.8 | 13.92 | 3.069 |  |  |  |  |
| 1.25 | 8.245 | 3.849 | 4.964 |  |  |  |  |
| 3.49 | 12.668 | 10.651 | 10.185 |  |  |  |  |
| 1.348 | 4.659 | 1.556 | 6.996 |  |  |  |  |
| 0.94 | 5.345 | 11.44 | 5.577 |  |  |  |  |
| 0.962 | 10.258 | 1.878 | 1.459 |  |  |  |  |
| 1.374 | 8.365 | 5.54 | 6.537 |  |  |  |  |
| 0.663 | 3.222 | 1.254 | 4.075 |  |  |  |  |
| 1.252 | 3.206 | 0.864 | 2.776 |  |  |  |  |
| 1.113 | 9.296 | 1.205 | 2.832 |  |  |  |  |
| 0.596 | 3.837 | 1.646 | 2.864 |  |  |  |  |
| 1.006 | 11.583 | 2.527 | 20.727 |  |  |  |  |
| 6.802 | 36.96 | 26.4 | 6.637 |  |  |  |  |
| 1.568 | 13.997 | 1.671 | 4.086 |  |  |  |  |
| 2.71 | 33.12 | 6.24 | 9.001 |  |  |  |  |
| 1.233 | 5.263 | 1.99 | 10.982 |  |  |  |  |
| 4.392 | 17.28 | 9.12 | 4.745 |  |  |  |  |
| 1.988 | 12.974 | 1.461 | 3.213 |  |  |  |  |
| 0.832 | 4.473 | 1.908 | 3.213 |  |  |  |  |
| 0.648 | 2.85 | 0.887 | 2.664 |  |  |  |  |
| 5.888 | 8.16 | 6.24 | 5.858 |  |  |  |  |
| 0.534 | 9.12 | 1.44 | 1.664 |  |  |  |  |
| 1.607 | 2.88 | 10.08 | 3.53 |  |  |  |  |
| 2.152 | 36.96 | 4.8 | 3.995 |  |  |  |  |
| 0.492 | 2.88 | 2.88 | 2.531 |  |  |  |  |
| 3.46 | 3.84 | 1.92 | 3.156 |  |  |  |  |
| 1.241 | 4.8 | 1.92 | 1.768 |  |  |  |  |
| 1.02 | 21.6 | 3.36 | 2.646 |  |  |  |  |
| 1.235 | 12 | 3.36 | 4.534 |  |  |  |  |
| 0.685 | 1.92 | 0.96 | 1.352 |  |  |  |  |
| 0.734 | 1.92 | 0.96 | 1.476 |  |  |  |  |
| 1.216 | 7.68 | 1.44 | 2.424 |  |  |  |  |
| 0.736 | 4.32 | 3.84 | 3.487 |  |  |  |  |
| 0.556 | 1.44 | 0.48 | 0.693 |  |  |  |  |
| 0.413 | 1.44 | 0.48 | 1.389 |  |  |  |  |
| 0.738 | 0.96 | 0.96 | 1.812 |  |  |  |  |
| 0.66 | 2.88 | 1.92 | 3.052 |  |  |  |  |
| 1.181 | 25.92 | 4.32 | 6.988 |  |  |  |  |
| 3.24 | 3.84 | 1.92 | 3.319 |  |  |  |  |
| 0.474 | 0.96 | 0.48 | 1.172 |  |  |  |  |
| 0.289 | 2.88 | 1.257 | 7.392 |  |  |  |  |
| 1.146 | 6.157 | 2.226 | 10.727 |  |  |  |  |
| 0.224 | 2.181 | 1.671 | 1.717 |  |  |  |  |
| 0.639 | 2.88 | 3.36 | 3.178 |  |  |  |  |
| 0.448 | 2.629 | 2.724 | 2.089 |  |  |  |  |
| 0.62 | 1.76 | 6.541 | 3.266 |  |  |  |  |
| 2.328 | 7.2 | 4.32 | 5.732 |  |  |  |  |
| 0.781 | 3.297 | 1.775 | 7.48 |  |  |  |  |
| 0.315 | 1.054 | 0.887 | 2.018 |  |  |  |  |
| 3.371 | 6.404 | 4.547 | 7.841 |  |  |  |  |
| 0.537 | 2.275 | 2.746 | 5.807 |  |  |  |  |
| 0.615 | 4.436 | 1.175 | 3.308 |  |  |  |  |
| 0.436 | 3.043 | 1.976 | 1.769 |  |  |  |  |
| 0.709 | 2.4 | 1.44 | 5.025 |  |  |  |  |
| 0.503 | 4.65 | 1.073 | 6.326 |  |  |  |  |
| 0.79 | 1.889 | 0.822 | 2.928 |  |  |  |  |
| 0.71 | 2.88 | 0.48 | 1.461 |  |  |  |  |
| 0.564 | 1.44 | 1.44 | 1.462 |  |  |  |  |
| 0.412 | 2.88 | 0.96 | 2.846 |  |  |  |  |
| 1.346 | 0.96 | 1.92 | 1.155 |  |  |  |  |
| 1.023 | 8.718 | 3.58 | 5.076 |  |  |  |  |
| 0.62 | 4.32 | 2.88 | 2.214 |  |  |  |  |
| 1.182 | 1.144 | 1.095 | 2.65 |  |  |  |  |
| 0.685 | 2.4 | 7.68 | 3.208 |  |  |  |  |
| 0.331 | 5.171 | 2.202 | 2.609 |  |  |  |  |
| 0.7 | 2.901 | 1.652 | 2.559 |  |  |  |  |
| 1.788 | 1.919 | 0.769 | 1.872 |  |  |  |  |
| 0.305 | 2.562 | 1.203 | 1.58 |  |  |  |  |
