# Supplementary material for: Naturally acquired antibodies against 4 Streptococcus pneumoniae serotypes in Pakistani adults with type 2 diabetes mellitus
Source: PLoS One. 2024 Aug 9;19(8):e0306921. doi: 10.1371/journal.pone.0306921 (PMC11315336; doi:10.1371/journal.pone.0306921)
Supplement: S3 Table — (DOCX) [file pone.0306921.s003.docx]

| 19F OPA titer | 18C OPA titer | 9V OPA titer |
| --- | --- | --- |
| 14 | 131 | 33 |
| 18 | 2 | 6 |
| 15 | 2 | 33 |
| 4 | 2 | 10 |
| 6 | 2 | 20 |
| 2 | 2 | 19 |
| 2 | 2 | 27 |
| 5 | 2 | 11 |
| 7 | 6 | 9 |
| 8 | 2 | 2 |
| 2 | 6 | 5 |
| 9 | 13 | 20 |
| 8 | 14 | 28 |
| 16 | 45 | 69 |
| 6 | 9 | 47 |
| 10 | 2 | 52 |
| 2 | 8 | 2 |
| 26 | 2 | 7 |
| 6 | 11 | 8 |
| 2 | 2 | 9 |
| 2 | 6 | 13 |
| 34 | 6 | 10 |
| 2 | 5 | 58 |
| 2 | 2 | 2 |
| 14 | 37 | 35 |
| 10 | 22 | 23 |
| 24 | 2 | 2 |
| 31 | 15 | 96 |
| 47 | 50 | 58 |
| 65 | 7 | 2 |
| 4 | 7 | 12 |
| 51 | 9 | 19 |
| 43 | 10 | 10 |
| 2 | 74 | 22 |
| 7 | 8 | 17 |
| 2 | 2 | 2 |
| 13 | 19 | 13 |
| 2 | 5 | 15 |
| 20 | 77 | 82 |

**S3 Table.** Serotype-specific opsonic titer values of participants
