# Supplementary material for: Naturally acquired antibodies against 4 Streptococcus pneumoniae serotypes in Pakistani adults with type 2 diabetes mellitus
Source: PLoS One. 2024 Aug 9;19(8):e0306921. doi: 10.1371/journal.pone.0306921 (PMC11315336; doi:10.1371/journal.pone.0306921)
Supplement: S4 Table — (DOCX) [file pone.0306921.s004.docx]

| DM-19F OPA | DM-9V OPA | DM-18C OPA | NDM-19F OPA | NDM-9V OPA | NDM-18C OPA |
| --- | --- | --- | --- | --- | --- |
| 14 | 131 | 33 | 14 | 37 | 35 |
| 18 | 2 | 6 | 10 | 22 | 23 |
| 15 | 2 | 33 | 24 | 2 | 2 |
| 4 | 2 | 10 | 31 | 15 | 96 |
| 6 | 2 | 20 | 47 | 50 | 58 |
| 2 | 2 | 19 | 65 | 7 | 2 |
| 2 | 2 | 27 | 4 | 7 | 12 |
| 5 | 2 | 11 | 51 | 9 | 19 |
| 7 | 6 | 9 | 43 | 10 | 10 |
| 8 | 2 | 2 | 2 | 74 | 22 |
| 2 | 6 | 5 | 7 | 8 | 17 |
| 9 | 13 | 20 | 2 | 2 | 2 |
| 8 | 14 | 28 | 13 | 19 | 13 |
| 16 | 45 | 69 | 2 | 5 | 15 |
| 6 | 9 | 47 | 20 | 77 | 82 |
| 10 | 2 | 52 | 10 | 22 | 23 |
| 2 | 8 | 2 | 24 | 2 | 2 |
| 26 | 2 | 7 | 31 | 15 | 96 |
| 6 | 11 | 8 | 47 | 50 | 58 |
| 2 | 2 | 9 |  |  |  |
| 2 | 6 | 13 |  |  |  |
| 34 | 6 | 10 |  |  |  |
| 2 | 5 | 58 |  |  |  |
| 2 | 2 | 2 |  |  |  |

**S4 Table.** Serotype-specific opsonic titer values those with and without type 2 diabetes
