# Supplementary material for: Naturally acquired antibodies against 4 Streptococcus pneumoniae serotypes in Pakistani adults with type 2 diabetes mellitus
Source: PLoS One. 2024 Aug 9;19(8):e0306921. doi: 10.1371/journal.pone.0306921 (PMC11315336; doi:10.1371/journal.pone.0306921)
Supplement: S5 Table — (DOCX) [file pone.0306921.s005.docx]

| DM-PspA IgG (OD450)  **S5 Table.** PspA IgG (OD 450) values of those with and without type 2 diabetes | NDM-PspA IgG (OD450) |
| --- | --- |
| 0.323 | 0.282 |
| 0.365 | 0.298 |
| 0.401 | 0.392 |
| 0.361 | 0.351 |
| 0.315 | 0.353 |
| 0.459 | 0.253 |
| 0.325 | 0.364 |
| 0.286 | 0.208 |
| 0.431 | 0.372 |
| 0.379 | 0.415 |
| 0.378 | 0.422 |
| 0.497 | 0.352 |
| 0.366 | 0.343 |
| 0.303 | 0.215 |
| 0.324 | 1.2858 |
| 0.418 | 0.6368 |
| 0.266 | 0.5818 |
| 0.451 | 1.2148 |
| 0.412 | 0.7538 |
| 0.201 | 1.2168 |
| 0.137 | 1.0078 |
| 0.404 | 1.7158 |
| 0.313 |  |
| 0.3 |  |
| 0.269 |  |
| 0.496 |  |
| 0.268 |  |
| 0.319 |  |
| 0.372 |  |
| 0.538 |  |
| 0.345 |  |
| 0.333 |  |
| 0.314 |  |
| 0.39 |  |
| 1.337 |  |
| 1.041 |  |
| 0.377 |  |
| 1.149 |  |
| 1.186 |  |
| 0.522 |  |
| 1.142 |  |
| 1.212 |  |
| 0.812 |  |
| 0.831 |  |
| 1.627 |  |
| 0.279 |  |
| 1.390 |  |
| 1.534 |  |
| 1.312 |  |
| 1.263 |  |
| 1.029 |  |
| 0.287 |  |
| 0.267 |  |
| 1.032 |  |
| 0.854 |  |
| 1.087 |  |
| 0.776 |  |
| 0.382 |  |
| 0.993 |  |
| 0.993 |  |
| 1.037 |  |
| 0.957 |  |
| 1.024 |  |
| 0.951 |  |
| 0.670 |  |
| 0.791 |  |
| 1.454 |  |
| 1.305 |  |
